# Ultra-fast adsorption of radioactive technetium (^99m^Tc) by using mining waste clay samples, Abu-Tartur, Egypt

**DOI:** 10.1038/s41598-023-42757-z

**Published:** 2023-09-23

**Authors:** Ahmed Saleh Ahmed, Walaa Ali Hassan, Mohamed Abdel-Moneim Mohamed, Ezzat Abdalla Ahmed, Nagih M. Shaalan, Mostafa Ragab Abukhadra

**Affiliations:** 1 Radiation Therapy and Nuclear Medicine Department, South Egypt Cancer Institute Assiut University, Asyût, Egypt; 2 Geology Department, Faculty of Science, Assiut University, Asyût, Egypt; 3 Department of Physics, College of Science, King Faisal University, 31982 Al-Ahsa, Saudi Arabia; 4 Physics Department, Faculty of Science, Assiut University, Asyût, 71516 Egypt; 5 Materials Technologies and Their Applications Lab, Geology Department, Faculty of Science, Beni-Suef University, Beni-Suef, Egypt

**Keywords:** Environmental sciences, Environmental chemistry

## Abstract

In this study, we have opened a great route to fabricate a high-performance nanocomposite for various functional applications based on the composite of a natural stone. A clay sample (black shale (B.Sh)) was collected from the Abu-Tartur area in Egypt. The black shale was organically modified with organic materials in our laboratory, which is called organo-black shale (O-B.Sh). The samples were characterized by XRD, FTIR, SEM, and XRF. These techniques confirmed that the samples have multi-oxide phases with approximately SiO_2_ at 54.1%, Al_2_O_3_ at 24.73%, Fe_2_O_3_ at 6.02%, K_2_O at 1.12%, MgO at 1.09%, and Na_2_O of 0.09%, as calculated by XRF. The two samples were applied to the adsorption processes of the radioactive technetium materials, which have been used for the medical treatment of the cancer institute of Upper Egypt. The adsorption processes were performed at various concentrations of the radioactive material and various amounts of clay samples. The as-collected B.Sh sample showed an adsorption activity of 65%, however, the organically modified materials showed a high adsorption rate toward technetium reaches to 100% in a very short time and without any further process. The present collected materials are very promising to withdraw the radioactive materials from the saline solution to save human and environmental health. We believe these multi-compound composites may open a new approach for creating new fabric composites with high performance for various applications.

## Introduction

Organoclays have important practical applications as adsorbents of inorganic and organic pollutants. The traditional raw materials for the synthesis of organoclays are phyllosilicates with the expanding structural cell of the smectite group, such as montmorillonite.

Black shale tremendous of smectite reaches up to (85.1%) forming bentonite which is recorded for the first time. Bentonite is characterized by excellent adsorption capacity for cationic pollutants but is usually an ineffective adsorbent for anionic contaminants. Huge studies have shown, however, that the adsorptive capacity of smectite for anionic radionuclides can be substantially improved by replacing the natural inorganic interlayer cations with quaternary alkylammonium cations (QAAC) in the form [RN(CH3)_3_]^+^, where R is an alkyl or aromatic hydrocarbon^1,2^.

**Figure 1:**
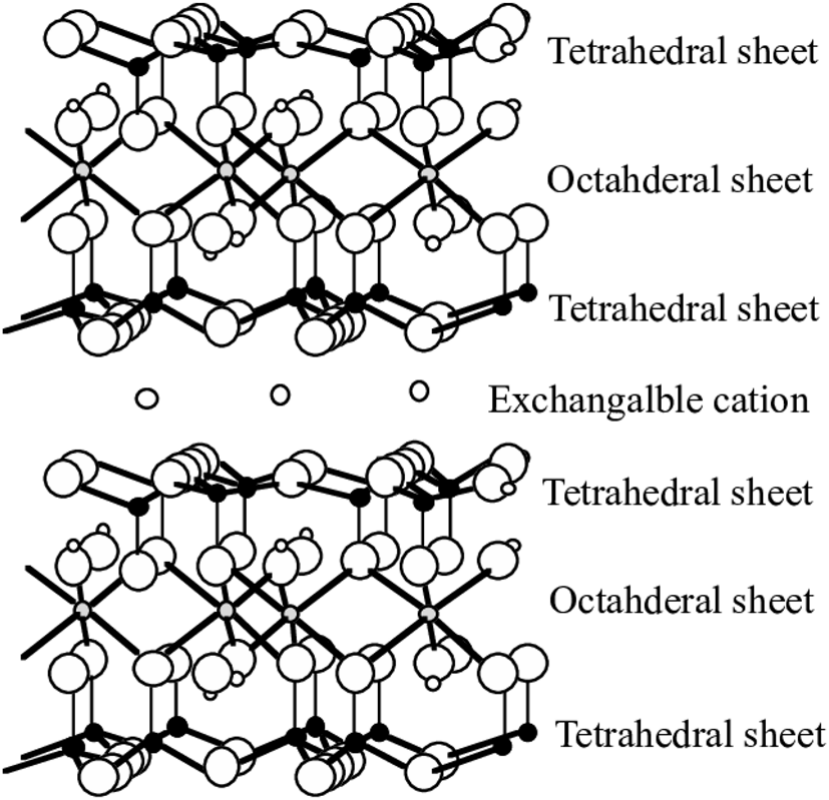
General Structure of Smectite^3^.

Smectite minerals are phyllosilicates with a 2:1 structure formed by stacking multiple layers of one octahedral layer between two tetrahedral ones. Understanding the structural formula of smectite minerals is basic to predicting its physicochemical properties, depending on the location of cation substitution in its 2:1 layer (Fig. 1)^3^.

Smectite layers have numerous isomorphic substitutions on both the tetrahedral (mainly Al^3+^ and secondarily Fe^3+^, instead of Si^4+^) and octahedral positions, as well as vacancies in the octahedral layer, giving rise to a layer charge. This layer charge is compensated by cations (Mg^2+^, Ca^2+^, Na^+^, K^+^) in the interlayer space connecting adjacent layers, are hydrated to different extents, and can exchange with cations from external solution.

The medicinal use of metastable ^99m^Tc causes the release of a substantial amount of ^99^Tc into the environment^4^. Thus, technetium is considered to be one of the important hazardous elements in high-level radioactive waste. ^99^Tc is suspected to cause lung cancer or other sickness and is more concerning than the current level of typical Tc ingestion through water or food. The control of environmental pollution by ^99^Tc is therefore considered a primary task. It is essential to develop reliable and cost-effective methods to remove ^99^Tc from a variety of vehicles, ranging from high-level liquid waste to low levels of radioactivity in groundwater. Many researchers have examined the immobilization of ^99^Tc, as well as its adsorption from contaminated groundwaters using various adsorbent materials^5–7^.

In this study, we have collected waste black shale stones from the Abu-Tartur phosphate mine area in Egypt. The B.Sh were used as collected and they have undergone organic treatment. Then we applied these materials, as low-cost-effective materials, for the removal of ^99^Tc from the salt solution, which was used in the medical diagnosis. The as-collected and organically treated stones are characterized by XRD, FTIR, and SEM techniques. The adsorption properties of ^99^Tc on these natural materials are investigated for various concentrations of ^99^Tc and different adsorbent technetium.

## Experimental details

### Materials

All commercially available reagents were of analytical grade and were used without further purification. Technetium (^99m^Tc and ^99^Tc) was eluted with sodium chloride solution using normal saline (0.9% NaCl) from a ^99^Mo/^99m^Tc generator used in nuclear medicine (Department of Radiotherapy and Nuclear Medicine, South Egypt Cancer Institute, Assiut University). The ^99^Mo was transformed into ^99m^Tc (87.6% of ^99^Mo disintegrations) and ^99^Tc (12.4% of ^99^Mo disintegrations). Both ^99m^Tc and ^99^Tc were assumed to be in the pertechnetate form (TcO_4_)^−1^. Clay samples, namely, Black shale: B.Sh) are collected from the Abu-Tartur area, the western desert of Egypt. Cetyltrimethylammonium bromide (CTAB) with a chemical formula of C_19_H_42_BrN and molecular weight of 364.45 g/mol as the surfactant was purchased from Alpha Chemika, India.

### Organoclay (adsorbent) preparation

20 g of clay was added to 1000 mL of distilled water and allowed to swell and homogenize for 24 h at room temperature using a magnetic stirrer. Then it was gradually added to an aqueous solution of 10 g CTAB dissolved in 500 mL H_2_O. The mixture was vigorously stirred for 24 h at 60 °C. afterward, the clay suspension was filtered and repeatedly washed with distilled water. Then the filter cack was dried in an oven at 80 °C, ground to 200 mesh, and kept in a sealed bottle. The collected sample of B.Sh is organically modified (O-B.Sh).

### Materials characterizations

XRD patterns were recorded for 2θ of 5 and 50° at a scanning speed of 3°/min using a Philips PW 1700 diffractometer with a copper target and Cu-K α radiation of λ = 1.54056 Å. Fourier transform infrared spectra (FTIR) using KBr pressed disk technique was gained on a Nicolet spectrophotometer (model: 6700). The spectra were examined for each measurement over the spectral range of 400–4000 cm^−1^ with a resolution of 1 cm^−1^. The surface morphology of clay and organoclay was carried out using Scanning Electron Microscope (JEOL, JSM-5400 LV). The aliquot activity of the supernatant was measured using CII Capintec (CRC-25R) gamma counter at the same time to avoid time correction (due to the short half-life of ^99m^Tc) in further calculations.

### Adsorption measurements

The batch experiments were performed by 0.5 g of sorbent added to 2 mL of NaCl salt solution with a known concentration of ^99m^Tc into a polypropylene centrifuge tube. Subsequently, the liquid phases were separated from the solid phases by centrifugation for 5 min at 3000 rpm. The aliquot activity of the supernatant was measured using CII Capintec (CRC-25R) gamma counter at the same time to avoid time correction (due to the short half-life of ^99m^Tc) in further calculations. The batch experiment and ^99m^Tc measurements were carried out by Atomlab Dose Calibrator (Atomlab 500) at the Department of Radiotherapy and Nuclear Medicine, South Egypt Cancer Institute, Assiut University.

## Results and discussion

### Characterization of clay and organoclay adsorbents

**Figure 2:**
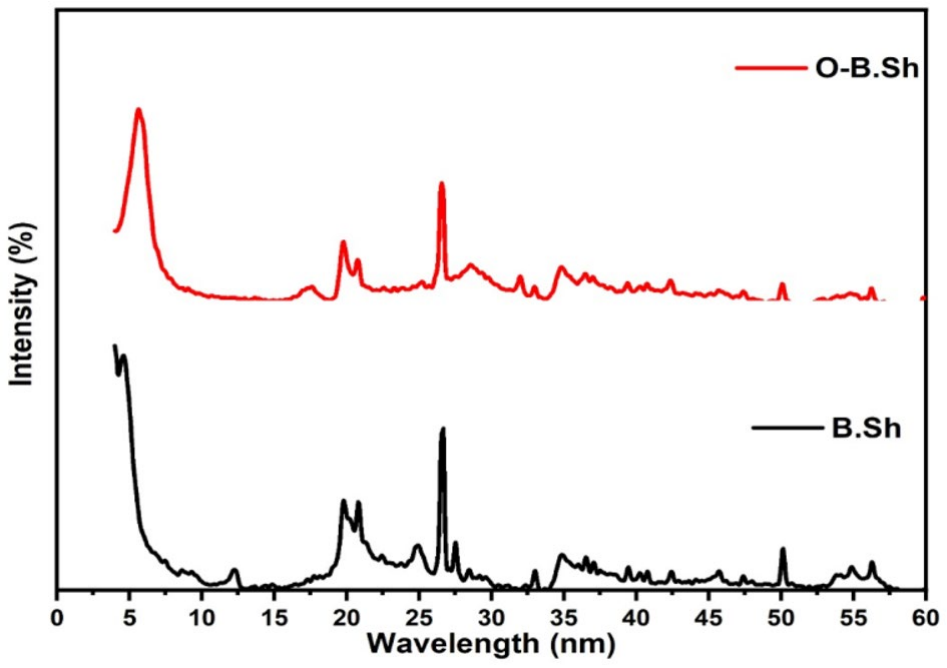
X-ray diffraction of B.Sh (Black Shale) and O-B.Sh (Organo-black shale).

XRD patterns of B.Sh and O-B.Sh samples are given in Fig. 2. In B.Sh, a remarkable decrease in the intensity of the main peak (2*θ* = 5.67°) belonging to bentonite-rich clay and appeared two new peaks in 2*θ* = 4.9° and 5.02°for the organically modified sample indicating two different arrangements of CTAB interlayer galleries of clay^8^. As shown from the forgoing results, with the addition of surfactant, the basal spacing of the resultant organoclay increases indicating the location of CTA^+^ ions between layers of clay mineral and which leads to a decrease of the hydration water content. Thus, the surface property changes from hydrophilic to hydrophobic. As known, the amount of added surfactant lead to interlayer expansion of smectite ^9,10^. XRD for irradiated black shale revealed there was no observable change of the structure of black shale after irradiation, but an evident decrease in the intensity of clay minerals content.

The chemical analysis was measured according to (EN196–2.2:2005) and (ASTM C114) by using X-ray fluorescence apparatus (XRF), (Model ARL 9900 by Thermo Scientific). for the investigated as withdrawn materials referring to the existence of the SiO_2_ of 54.1%, Al_2_O_3_ of 24.73%, Na_2_O of 0.09%, K_2_O of 1.12%, Fe_2_O_3_ of 6.02%, and MgO of 1.09%. The XRF data confirmed the multi-oxide phase for the investigated black shale. The most dominant oxide is SiO_2_ followed by Al_2_O_3_ and Fe_2_O_3_. The other rest oxide comes with low concentrations, or we can say doping level. The chemical formula calculated for the black shale (Smectite) is [Na_0.003_K_0.01_Fe_0.25_ Mg_0.09_ Al_0.85_ (Al_0.86_Si_3.14_ O_10_) (OH)_2_. 0.7(H_2_O)].

**Figure 3:**
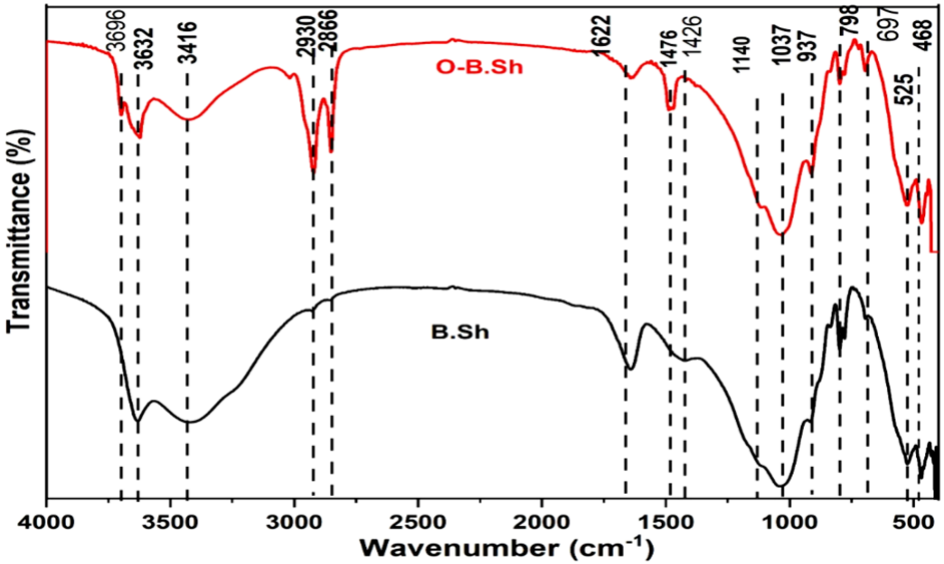
FT-IR spectrum of B.Sh and O-B.Sh.

The FTIR spectra of unmodified clay and the modified clay samples by adsorption of CTAB are shown in Fig. 3. Organoclay sample (O-B.Sh) shows the peaks of the pure clay minerals, new characteristic peaks appear at 2923, 2851, 2920, and 2851 cm^−1^ corresponding to the –CH_2_ asymmetric and –CH_2_ symmetric stretching vibrations respectively (Fig. 3). These bands are absent in the pure clay sample (B.Sh). IR spectrum which indicates the incorporation of the surfactant in organoclay. According to^11^, the bands are very narrow, and the variation of their intensity increases with increasing initial surfactant content, indicating the intercalation of a higher amount of surfactant in clay with increasing the initial amount of surfactant.

Organoclay corresponds to a linear direct cationic exchange of the exchangeable alkali cations by the cationic surfactant. As shown in Fig. 3, when the alkyl intercalated into clays, a broad band at 1489 and 1488 cm^−1^ splits with the addition of surfactant^11^. For the –CH_2_ rocking modes, with the CTAB adding, two well-resolved vibration bands at 798 and 797 cm^−1^ were observed, similar to –CH_2_ scissoring. Splitting of the methylene scissoring and rocking modes not only relates to surfactant loading but also relates to the chemical structure of the used surfactants^11^. According to^12^, “spectral hydration features in bentonite have been attributed to the structural OH in the octahedral layer, water adsorbed on the clay external surfaces, and water adsorbed in the internal regions.” The property of these interlayer water molecules is greatly dependent on the moisture level and the interlayer cation. Bands between 3600 and 4000 cm^−1^ were attributed to the Al_2_OH group of the octahedral layer from the IR spectra of raw materials and organoclays (Fig. 3). A broadband around between 3417 and 3424 cm^−1^ observed in the raw materials and organoclay samples were ascribed to the overlapping symmetric and asymmetric hydroxyl group stretching vibration of water molecules on the external layer^12^.

**Figure 4:**
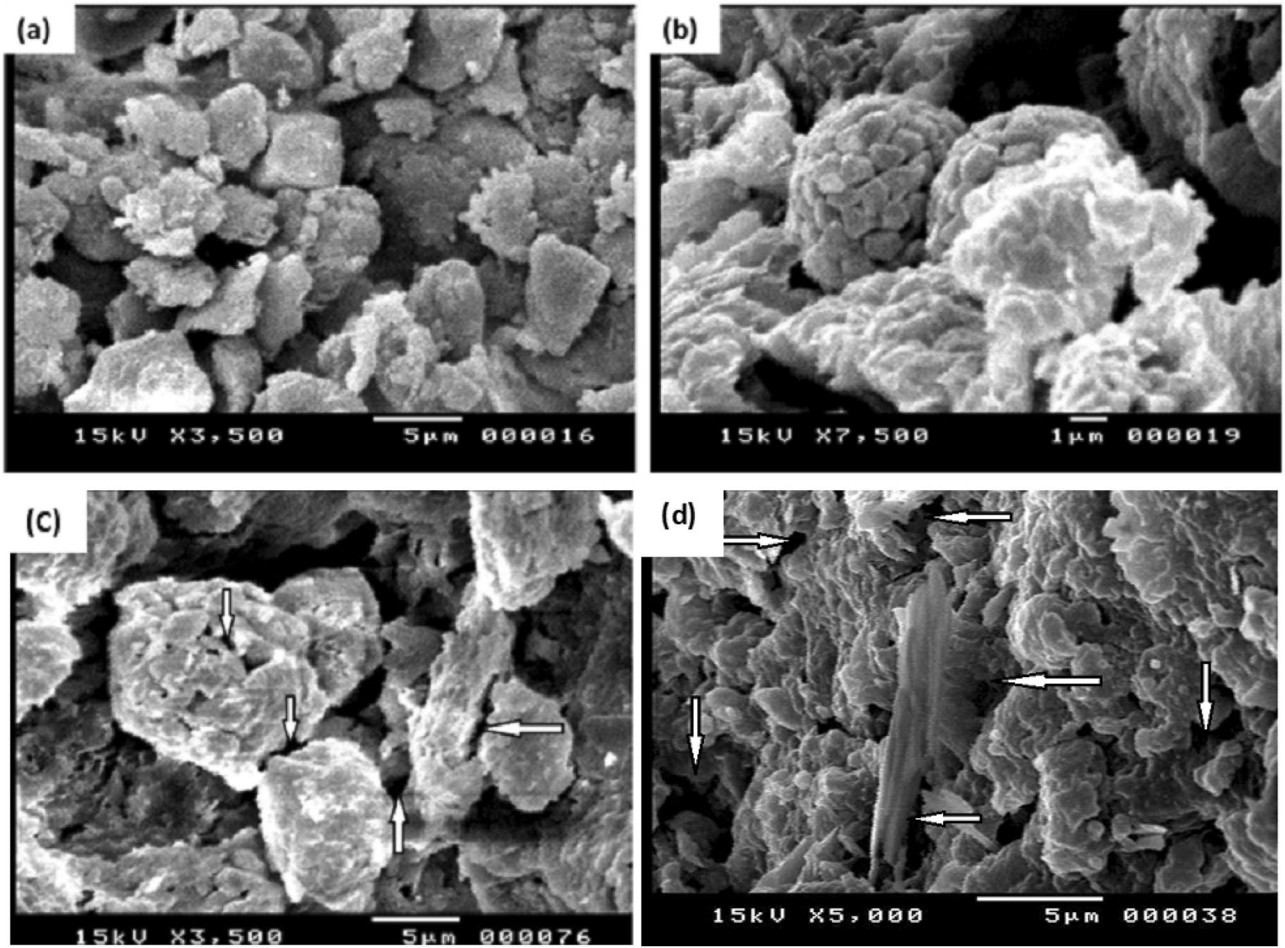
SEM images of (**a**) B.Sh, (**b**) O-B.Sh, (**c**) irradiated B.Sh, and (**d**) irradiated O-B.Sh (white arrows in Figs. c and d referred to tensile fractures and fibril structure.

The surface morphology of clay and organoclay samples is given in Fig. 4. Both unmodified and modified clays have an uneven structure with non-uniform size distribution. It can be seen that the unmodified clay (Fig. 4a) has massive, aggregated morphology, and some large flakes were observed in some instances. Compared with the morphology of the clay, there are many small flakes with severely crumpled structures in organoclay samples (Fig. 4b). Due to the increase of basal spacing in organoclays, more voids are seen.

The significant improvements in tensile properties of irradiated B.Sh and O.B.Sh samples compared with these nonirradiated samples are supported by SEM micrographs^13^. The morphology of the irradiated samples (Fig. 4c and d) is significantly different from that of nonirradiated samples (Fig. 4a and b). The irradiated samples are characterized by the formation of tensile fracture and fibril-like structure (as indicated by the white arrows). Stretching and elongation of matrix in the irradiated samples shows a higher resistance toward failure.

**Figure 5:**
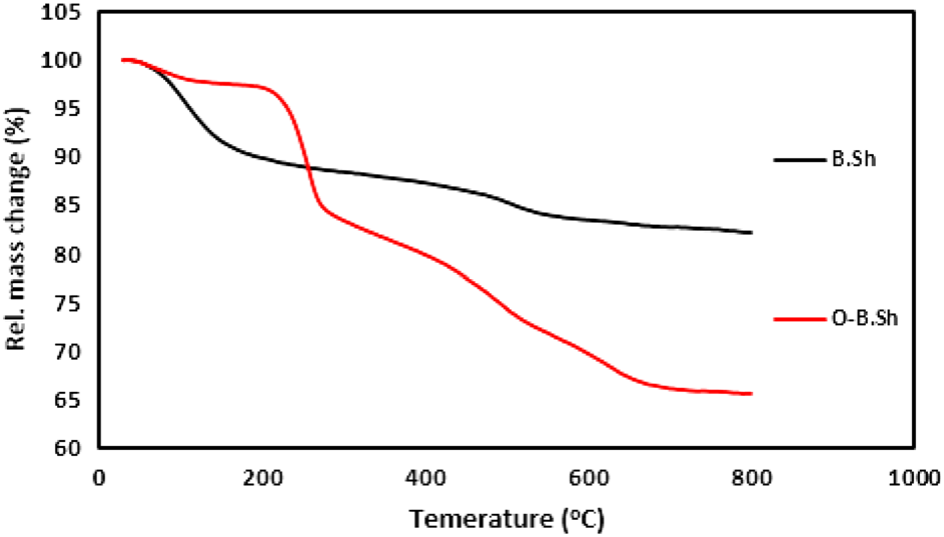
TGA analysis of B.Sh and O-B.Sh.

Thermal stability plays an essential role in determining both technological applications and processing conditions of organoclays. The thermal decomposition expressed in terms of weight loss as a function of temperature of black shale and organo-black shale are given in Fig. 5. The results indicate that the organo-black shale have a higher thermal stability than black shale. For the black shale an initial weight loss between 39 and 164 °C is observed. The second decomposition was between 164 and 484 °C, and the third one was between 484 and 700 °C. The total weight loss calculated is 17.76%. According to^14^ the montmorillonite differential thermal curve divided in two parts; (a) the free water and interlayer water reign in the temperature range 100–200 °C (b) the structural water (bonded OH that undergoes dehydroxylation) region in the temperature range 500–1000 °C.

For organo-black shale the first decomposition measured at temperature between 164 and 290 °C, the second between 290 and 460 °C, and the third one between 460 and 800 °C. The total weight loss calculated is 33.76%. According to^15^ the decomposition of the organoclay divided into four parts; (a) the free water region in the temperature below 200 °C (b) the region where organic substances evolve in the temperature range 200–500 °C (c) the structural water region in the temperature range 500–800 °C and (d) the region between 800 and 1000 °C. Where organic carbon reacts with inorganic oxygen (combustion reaction) at temperature 800 °C.

### Technetium removal

To evaluate the adsorption capacity and adsorption isotherms, the effect of contact time, initial concentration of ^99m^Tc, and sorbent dosage were examined.The uptake percent or the removal efficiency (*R%*), as well as the adsorption capacity (qe), were calculated by the following Eqs. (1 and 2):

1 $$R\%= {(C}_{0}-Ce)/{C}_{0}*100$$

2 $${q}_{e} = V({C}_{0}-{C}_{e})/m$$

Where C_0_ is the initial concentration of ^99m^Tc, *C*_*e*_ is the concentration of ^99m^Tc at equilibrium, *V* (in L) is the volume of ^99m^Tc solution, and *m* (g) is the mass of sorbent.

**Figure 6:**
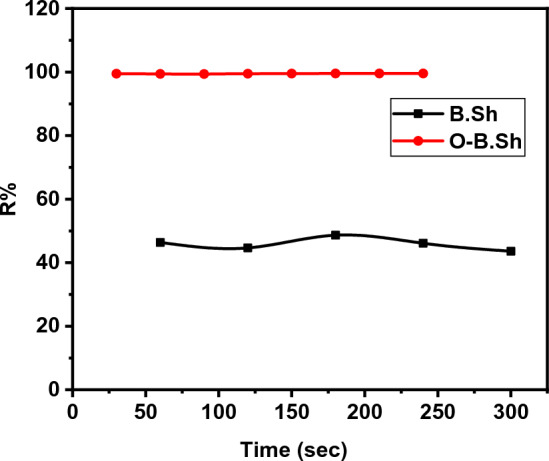
Effect of contact time on ^99m^Tc removal percent for of (B.Sh and O-B.Sh) samples.

To define the effect of contact time on the ^99m^Tc adsorption onto clay and organoclay samples, batch adsorption tests were carried out at different contact times ranging from 30 to 480 s using 0.5 g of each sorbent and initial activity 185 MBq, and the obtained results were presented in Fig. 6. In the case of unmodified clays as sorbents, the removal percent of ^99m^Tc increased from about 45% to about 50%. as a contact time increase from 30 to 90 s respectively. Using modified clay (organoclay) sorbent, R% reaches up to 100% at a contact time of 30 s. The faster initial uptake can be attributed to the presence of a large number of empty adsorption sites available; as the technetium is adsorbed onto the adsorption sites of the brick particles, the number of these sites decreases, and the slope flattens as the adsorption rate decreases^16^.

**Figure 7:**
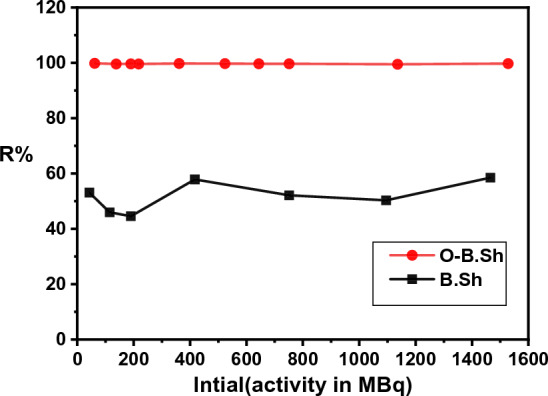
Effect of initial activity (MBq) on99mTc removal percent for (B.Sh and O-B.Sh) samples.

Figure 7 shows the calculated activity R% at different concentrations of ^99m^Tc (initial radioactivity). To examine the effect of initial activity, the adsorption processes are carried out at a different initial activity of ^99m^Tc between 37 to 1528 MBq at a constant sorbent dosage of 0.5 g and contact time of 3 min. The *R%* slightly fluctuated with different initial radioactivities, which reach a bout of 40–60% using B.Sh. adsorbent. However, the same amount of adsorbent was large enough to obtain 100% efficiency with various concentrations of initial radioactivity using O.B.Sh adsorbent.

**Figure 8:**
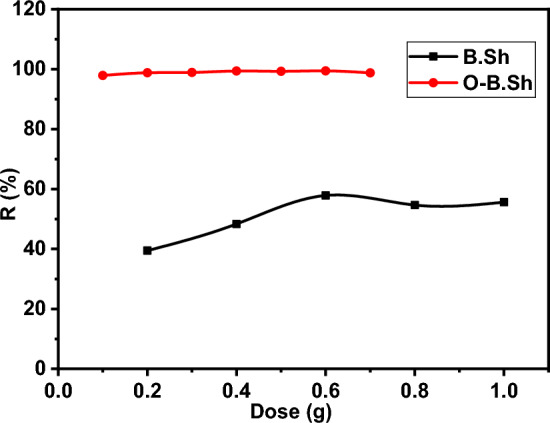
Effect of absorbent dosage on ^99m^Tc removal percent for (B.Sh and O-B.Sh) samples.

Based on the above data, we have studied the effect of the adsorbent amount on the percentage removal, R% of ^99m^Tc, as shown in Fig. 8. The adsorbent amount started from 0.1 to 1 g. For the as-collected material, the R% was recorded at 40% at a low amount of 0.2 g and enhanced up to 60% at 0.6 g. It showed a constant efficiency above this amount. The percentage removal of ^99m^Tc increase with increasing in the adsorbent dosage for the unmodified clay (0.2 to 0.6), while 0.1 g of modified organoclay was sufficient to remove up to 99% of ^99m^Tc. This can attribute to the increases in the surface area and the number of active sites of organoclay^17^.

### Isothermal and kinetic adsorption models

Various kinds of isotherms can be distinguished depending on the nature of the adsorbent and the interaction type. Two common isotherm models (Freundlich model and Langmuir model) were studied to detect the adsorption behavior of ^99m^Tc using black shale absorbent and their modified organoclays. Langmuir's isothermal model assumes that the adsorption takes place at specific homogeneous sites within the adsorbent with no lateral interaction between the sorbed molecules^18^.

In general, these models are characterized by certain constant values which describe the surface properties of the sorbents and their affinities toward cations and anions, and therefore, they can use to assess their adsorption capacities. The linear expression of Langmuir isotherm models^19^ can be represented by the following equations:

3 $$1/q_{e} = \, 1/\left( {K_{L} \cdot q_{max} } \right) \cdot \left( {1/Ce} \right) + 1/q_{max}$$

where *q*_max_ (MBq g^−1^) is the monolayer capacity of the adsorbent, and *K*_L_ (cm^3^ g^−1^) is the Langmuir constant. Langmuir isotherm can be expressed by a dimensionless constant called separation factor or equilibrium parameter (*R*_*L*_), defined by Weber and Chakkravorti equation^20^:

4 $$R_{L} = 1/\left( {1 + K_{L} C_{0} } \right)$$

Freundlich's isothermal model describes adsorption as taking place on a heterogeneous adsorbent surface. The linear form of the Freundlich isotherm model was represented by Eq. (5)^21^:

5 $$lnq_{e} = ln \, K_{F} + \, lnC_{e} * \, 1/n$$

where q_e_ (MBq g^−1^) is the amount of ^99m^TcO_4_^–^ activity adsorbed on the adsorbent at equilibrium, and *K*_F_ (cm^3^ g^−1^) and *n* are the Freundlich constants characteristic of the system. The value of *K*_F_ is related to the affinity of an adsorbent toward the investigated adsorbate and higher KF values indicate higher affinity. The adsorption process can be chemically and/or physically heterogeneous.

**Figure 9:**
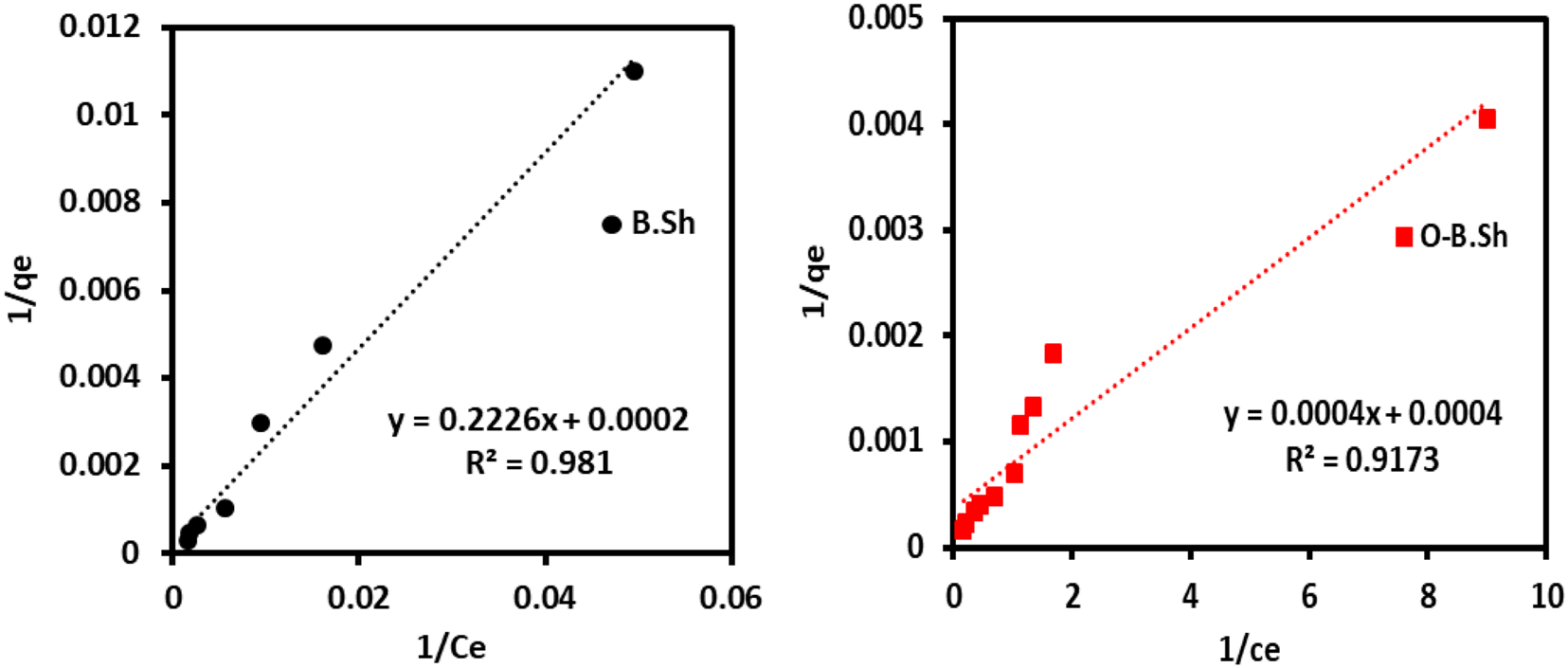
Langmuir adsorption isotherms obtained for ^99m^Tc uptake onto B.Sh and O-B.Sh.

**Figure 10:**
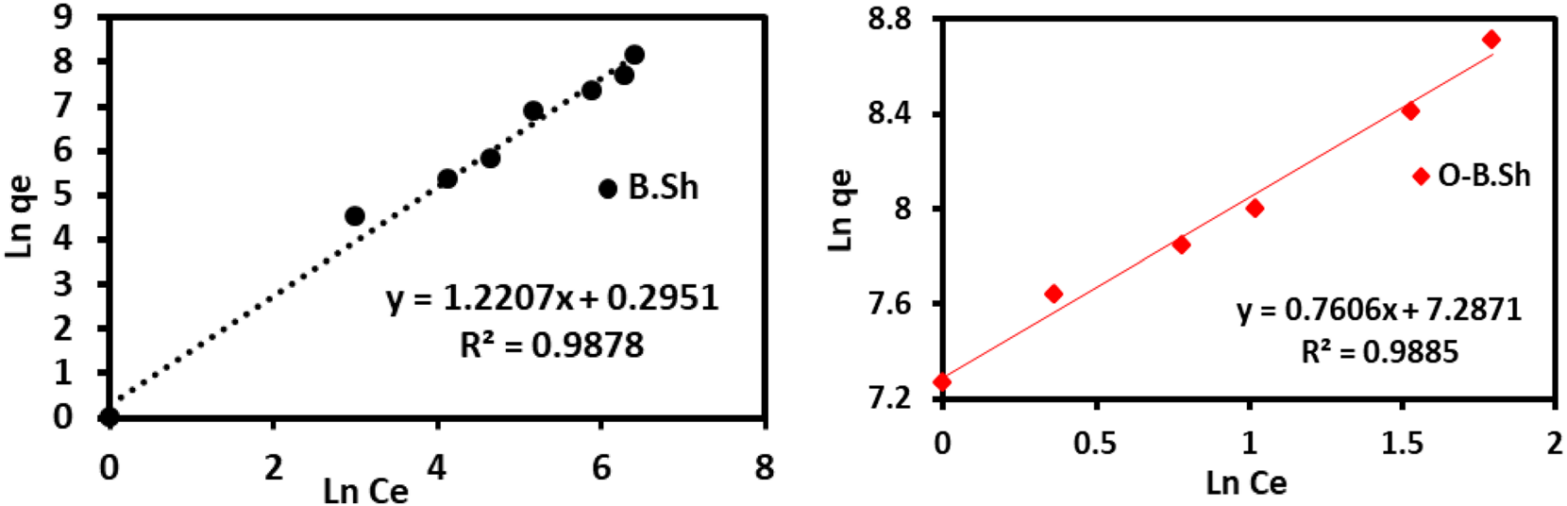
Freundlich adsorption isotherms were obtained for ^99m^Tc uptake for B.Sh and O-B.Sh.

In the present study, to apply the Langmuir and Freundlich isothermal models, the adsorption processes were carried out at initial activity 37 to 1528 MBq with a constant sorbent amount of 0.5 g and contact time of 3 min. To obtain the constants of the Langmuir and Freundlich isotherm models, the linear plots of Eqs. (3 and 5) are depicted in Fig. 9 for the Langmuir model and Fig. 10 for the Freundlich model. Consequently, from the slope and intercept of these linear plots, these constants and other parameters were calculated and tabulated in Table 1.

**Table 1:** Adsorption isotherm parameters of ^99m^Tc onto B.sh and O-B.Sh.

Model	Parameters	Sample	
		B.Sh	O-B.Sh
Freundlich	*1/n*	0.99	0.62
	*K*_*f*_	1.9	1550
	*R*^*2*^	0.977	0.98
Langmuir	*R*^*2*^	0.98	0.9171
	*Q*_*max*_	5000	2500
	*Kl*	0.000898	1
	*Rl*	0.00068–0.02276	0.150–0.473709

Using waste rocks absorbent of B.Sh, the coefficient of determination (*R*^*2*^) for the Langmuir isotherm was 0.98 (Fig. 9), Adsorption is considered favorable if the R_L_ value follows 0 < RL < 1; otherwise, it is considered unfavorable (RL > 1), linear (RL = 1) and irreversible (RL = 0). In the present study, the measured RL < 1 indicated the favorable adsorption of ^99m^Tc onto unmodified clay. These reflect the more suitability of the Langmuir isothermal model in describing the adsorption of ^99m^Tc onto unmodified clays than organoclays. On the other hand, using modified clay adsorbents O-B.Sh, the coefficient of determination (*R*^*2*^) of the Langmuir isotherms was 0.92, and *R*_L_ < 1.

As described in Eq. (5), values of *K*_F_ and *1/n* in the Freundlich isotherm model are calculated for the waste black shale and organoclays absorbents. For B.Sh, *K*_F_ calculated was 1.9, whereas 1550 for organoclays, as listed in Table 1. The Freundlich exponent values of n determine the intensity and feasibility of the adsorption process. If a value of *1/n less than 1.0*, it indicates a favorable (normal) adsorption, and if it is *greater than 1.0* indicates unfavorable (cooperative) adsorption. The values of *1/n* for both as collected and organoclays are less than 1.0 (between 0.62 and 0.99), indicating favorable normal adsorption. Moreover, the coefficient of determination in the Freundlich isotherm model for both adsorbent types is near unity, which is higher than that for the Langmuir isotherm. This result indicates that the equilibrium data of ^99m^Tc are well-fitted with the Freundlich isotherm model.

Adsorption is the process by which solute molecules attach to the surface of an adsorbent. The adsorption process is done in batch or column setup. During adsorption, two main processes are involved: physical (physisorption) and chemical (chemisorption). Physical adsorption is because of weak forces of attraction (van der Waals), while chemisorption involves the formation of a strong bond between the solute and the adsorbent with the transfer of electrons. Adsorption kinetics is a curve (or straight line) that represents the rate of retention or release of a solute from the aqueous environment to the solid phase interface at a given adsorbent dose, temperature, flow rate, and pH^22^. Kinetics is influenced by the surface complexity of the adsorbent, solute concentration, and flow^23^.

Pseudo-First order (PFO), Pseudo-Second order (PSO), Elovich, and Intra-particle (IP) models are some of the kinetics that foretells the adsorbent-adsorbate interaction. In every adsorption model, linear or nonlinear analysis of the kinetics is applied. The suitability of any model depends on the correlation coefficient (R^2^). To understand the detailed characteristics of the ^99m^Tc adsorption using a rock as collected and organoclays adsorbents, these three models were applied; however, the pseudo-second-order described the dynamics of the adsorption process well than others. Sometimes, for a complex system, the combination of two or more steps may be accountable for the overall rate^24^. The adsorption kinetic models are studied at a constant initial concentration (≃ 185 MBq) and adsorbent amount of 0.5 g with variable contact time between 30 and 180 s.

The formula for pseudo-second-order kinetics^25,26^, is generally employed in references in the form proposed by^27^ as Eq. (6):

6 $$t/q_{t} = \, 1/\left( {k_{2} q_{e}^{2} } \right) \, + \, t/qe$$

where q_e_ and q_t_ are the amounts of adsorption at equilibrium at a particular time *t*, in mg/g, respectively. k_2_ represents the overall rate constant for pseudo-second-order adsorption with the unit of MBq g^−1^ min^−1^. A linear plot will be achieved from the graph of t/qt versus time, t. Additionally, the initial adsorption rate *h* (MBq g^−1^ min^−1^) can be determined using Eq. (7)^28^:

7 $$h \, = \, k_{2} \cdot \, qe^{2}$$

**Figure 11:**
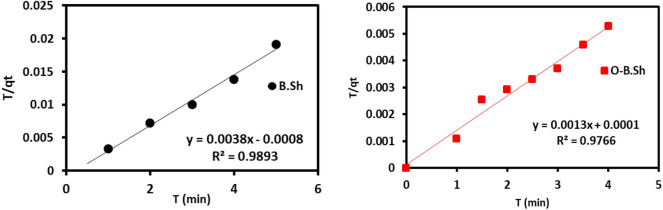
Pseudo second-order kinetic model of ^99m^Tc adsorption onto B.Sh and O-B.Sh.

The PSO kinetics is usually associated with the situation in which the rate of the adsorption process (seen as a kind of chemical reaction) controls the overall adsorption kinetics. The kinetic constants of adsorption were calculated from the slope and intersect of the linear relations, as shown in Fig. 11. The linear regression correlation coefficient (R^2^) values were compared to evaluate the best-fit model; the results are given in Table 2. The coefficient of determination (R^2^) values for pseudo-second-order are close to unity (between 0.98 and 1). In addition to the graphical harmony seen in Fig. 11, the pseudo-second-order model is the most suitable kinetic model for interpretation adsorption of ^99m^Tc onto both as collected and organoclay adsorbents.

**Table 2:** Parameters of Pseudo second-order kinetic model of ^99m^Tc adsorption onto B.sh and O-B.Sh.

Model	Parameters	Sample	
		B.Sh	O-B.Sh
Pseudo-second order	qe	263	769
	K2	0.02	0.017
	R^2^	0.99	0.98

## Conclusions

In summary, a natural nanocomposite of black shale clay was collected from a natural area in Egypt. The black shale was organically modified with organic materials. The collected sample has multi-oxide phases with approximately SiO_2_ of 54.1%, Al_2_O_3_ of 24.73%, Fe_2_O_3_ of 6.02%, K_2_O of 1.12%, MgO of 1.09%, and Na_2_O of 0.09%. The two samples were applied to the adsorption processes of the radioactive technetium materials, which have been used for medical treatment. The adsorption processes were performed at various concentrations of the radioactive material and various amounts of clay samples. The as-collected black shale sample showed an adsorption activity as high as 65%, however, the organically modified clay exhibited a high adsorption efficiency of 100% toward technetium in a very short time. The results exhibited that the present collected materials are very promising to remove the radioactive materials from the saline solution in a safe manner. Moreover, the results open show a new approach toward the fabrication of multi-compound composite with high performance toward various applications.

## Data Availability

The datasets used and/or analyzed during the current study available from the corresponding author on reasonable request.
